# Beyond Taxonomy: A Matrix–Trait–Function Framework for Predictive Selection of Non-*Saccharomyces* Yeasts in Food Fermentation

**DOI:** 10.3390/microorganisms14061378

**Published:** 2026-06-22

**Authors:** Nora Haring, Milan Chňapek, Blažena Drábová

**Affiliations:** Department of Biotechnology, Faculty of Biotechnology and Food Sciences, Slovak University of Agriculture in Nitra, Tr. A. Hlinku 2, 949 76 Nitra, Slovakia; qharing@uniag.sk (N.H.); milan.chnapek@uniag.sk (M.C.)

**Keywords:** non-*Saccharomyces* yeasts, food fermentation, strain specificity, microbial metabolism, aroma modulation, bioactive transformation, matrix-dependent fermentation

## Abstract

The growing diversity of food fermentation systems has intensified interest in non-*Saccharomyces* yeasts due to their broad metabolic capabilities and technological potential. However, current understanding of yeast functionality remains fragmented and frequently relies on taxonomy-centered classification, which often provides limited predictive value across fermentation systems. This review critically examines how strain-specific microbial traits, food matrix composition, and process conditions collectively shape fermentation performance across brewing, wine, cereal, plant-based, and functional fermentation systems. Particular emphasis is placed on key determinants of microbial functionality, including carbon metabolism, aroma biogenesis, acidification, enzymatic activity, microbial interactions, and transformation of food-associated bioactive compounds such as glycosides, phenolics, terpenes, and matrix-bound metabolites. The available evidence demonstrates that fermentation-relevant functionality cannot be reliably inferred from species identity alone because microbial performance is strongly modulated by strain variability and matrix-dependent environmental constraints. To address these limitations, this review proposes a matrix–trait–function framework that integrates microbial metabolic capabilities with food matrix characteristics and technological objectives to support a more predictive and application-oriented approach to yeast selection in food fermentation systems.

## 1. Introduction

The growing demand for fermented foods and beverages with improved sensory properties, reduced alcohol content, enhanced nutritional value, clean-label characteristics, and sustainability-oriented production has intensified interest in microbial diversity beyond conventional fermentation systems [1,2]. Although *Saccharomyces cerevisiae* remains the dominant industrial yeast due to its robust fermentative performance, predictable metabolism, and technological reliability, contemporary food fermentations increasingly require more targeted microbial strategies capable of delivering product-specific sensory, technological, and functional outcomes [3,4]. In this context, non-*Saccharomyces* yeasts have gained considerable attention owing to their broad metabolic capabilities and their potential to expand the technological versatility of food fermentations beyond ethanol production alone [5,6].

Unlike conventional fermentative systems primarily optimized for ethanol production and process reproducibility, non-*Saccharomyces* yeasts exhibit substantial strain-dependent variability in carbon metabolism, aroma biosynthesis, organic acid production, enzymatic activity, microbial interactions, and environmental adaptation [5,7]. These properties have positioned them as promising microbial tools for improving sensory complexity, modulating acidity, controlling ethanol formation, enhancing texture-related properties, and increasing process flexibility across a broad spectrum of fermented foods and beverages [2,8]. Consequently, their application increasingly extends beyond wine fermentation toward brewing, cereal fermentations, plant-based products, functional beverages, and mixed microbial systems [9,10,11].

Despite rapidly growing research interest, the current body of literature addressing non-*Saccharomyces* yeasts remains highly fragmented. Existing reviews are predominantly structured according to taxonomic classification, individual species characteristics, or sector-specific applications such as wine, beer, bakery, dairy, or plant fermentations [2,3]. While these approaches have substantially advanced understanding of yeast biodiversity and technological potential, they frequently provide descriptive overviews that insufficiently integrate the mechanistic determinants governing fermentation performance, including carbon metabolism, aroma precursor transformation, enzymatic activity, microbial interactions, stress adaptation, and environmental regulation of microbial functionality. As a consequence, fermentation-relevant outcomes are frequently interpreted through broad species-level descriptors that may provide only limited predictive value across different food matrices and technological settings.

Importantly, increasing evidence suggests that fermentation functionality cannot be reliably inferred from species identity alone because two distinct but interacting dimensions strongly shape microbial performance: strain specificity and matrix dependence. First, fermentation-relevant functionality frequently varies at the strain level, although the degree of variability may differ among species [5,12,13]. Strains belonging to the same species may exhibit markedly different behavior regarding sugar utilization, aroma production, organic acid metabolism, enzymatic activity, microbial competitiveness, and stress tolerance, thereby limiting the predictive value of broad species-level generalizations [5,12,13]. Consequently, species commonly described as “low-alcohol yeasts”, “aroma-enhancing yeasts”, or “acidifying yeasts” may not consistently display identical technological performance across strains or fermentation systems.

Second, fermentation performance is strongly influenced by food matrix composition, meaning that microbial functionality does not emerge solely from intrinsic metabolic potential but rather from interactions between microbial physiology and the physicochemical environment in which fermentation occurs [14,15,16]. Factors including sugar composition, nitrogen availability, oxygen transfer, pH, polyphenol content, water activity, and indigenous microbial ecology may substantially reshape metabolic responses and final product quality [15,17]. Consequently, the same yeast species—or even closely related strains—may exhibit markedly different technological behavior depending on fermentation substrate and process conditions.

Although strain specificity and matrix dependence represent conceptually distinct dimensions, they should not be interpreted independently because fermentation functionality ultimately emerges through their interaction. Strain-specific metabolic capabilities may be either enabled, constrained, or redirected by matrix composition and environmental conditions, resulting in context-dependent fermentation outcomes. This interaction represents an important yet frequently underexplored limitation of taxonomy-centered yeast classification and poses a major challenge for the predictive utilization of microbial diversity in food fermentation systems.

Beyond technological and sensory applications, increasing evidence indicates that selected non-*Saccharomyces* yeasts may additionally contribute to the transformation of food-associated bioactive compounds through enzymatic and metabolic activities [18,19]. Processes including glycoside cleavage, terpene liberation, modification of phenolic compounds, and release of matrix-bound metabolites have attracted growing attention, particularly in plant-derived and functional food fermentations [20,21]. Nevertheless, current evidence remains fragmented across food systems and microbial systems, limiting broader mechanistic understanding and practical translation.

**Figure 1:**
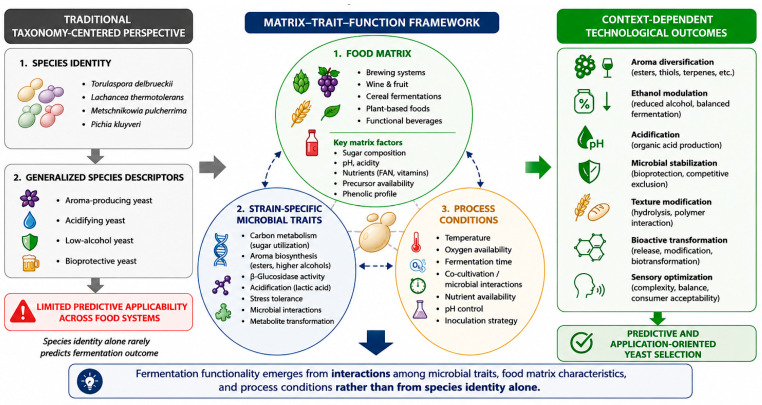
Matrix–trait–function framework governing non-*Saccharomyces* yeast functionality in food fermentation systems. The schematic illustrates a functionality-oriented perspective integrating food matrix characteristics, strain-specific microbial traits, and process conditions to explain context-dependent fermentation outcomes. Rather than relying solely on taxonomy-centered interpretation, the proposed framework conceptualizes fermentation functionality as an emergent property arising from dynamic interactions among matrix composition, microbial metabolism, and environmental constraints, thereby supporting a more predictive and application-oriented approach to yeast selection.

Unlike previous reviews predominantly organized around taxonomy, individual species characteristics, or sector-specific applications, the present review adopts a functionality-oriented perspective integrating food matrix composition, strain-specific microbial traits, and process conditions to better explain context-dependent fermentation performance. To conceptualize these interactions and address the limitations of taxonomy-centered interpretation, this review proposes a matrix–trait–function framework, in which fermentation functionality is interpreted as an emergent property arising from interactions among microbial metabolic traits, food matrix characteristics, and environmental constraints rather than from species identity alone. In contrast to conventional taxonomy-oriented selection strategies, this framework aims to provide a more predictive and application-oriented perspective for understanding and utilizing non-*Saccharomyces* yeast diversity across food fermentation systems (Figure 1).

The objective of this review is therefore not to provide another species-by-species overview of non-*Saccharomyces* yeasts but rather to critically examine how strain-specific microbial traits interact with food matrix composition and process conditions to determine fermentation functionality. Particular emphasis is placed on matrix-dependent functionality, key metabolic traits governing technological performance, and the emerging role of non-*Saccharomyces* yeasts in the transformation of food-associated bioactive compounds. By integrating microbial functionality with technological objectives, this review aims to provide a more predictive and application-oriented perspective for targeted yeast selection and fermentation design.

## 2. Materials and Methods

The present review was designed as a structured critical narrative review aimed at examining the functionality of non-*Saccharomyces* yeasts across diverse food fermentation systems, with particular emphasis on strain specificity, matrix dependence, metabolic functionality, and yeast-mediated transformation of food-associated bioactive compounds. Rather than providing a systematic species-by-species overview, the review adopted a functionality-oriented perspective focused on mechanistic interactions among microbial traits, food matrix composition, process conditions, and resulting technological outcomes.

Literature retrieval was conducted between January 2026 and May 2026 using the scientific databases Web of Science, Scopus, PubMed, and Google Scholar. Primary emphasis was placed on peer-reviewed studies published between 2022 and May 2026 to ensure contemporary relevance and reflect recent advances in non-*Saccharomyces* yeast research, particularly regarding strain specificity, food matrix interactions, metabolic functionality, and emerging technological applications. Earlier studies were included selectively when considered essential for conceptual background, mechanistic understanding, or historical context.

Searches were conducted using combinations of keywords, including non-*Saccharomyces* yeasts, food fermentation, yeast functionality, strain specificity, matrix-dependent fermentation, microbial metabolism, aroma formation, ethanol modulation, acidification, enzymatic activity, microbial interactions, bioactive transformation, phenolic transformation, glycoside cleavage, terpene release, and related combinations adapted to the scope of individual databases. Example search combinations included (“non-Saccharomyces yeast*” OR “non-conventional yeast*”) AND (“food fermentation” OR brewing OR wine OR sourdough OR plant-based fermentation). Publications were initially screened based on title and abstract relevance, followed by full-text evaluation. Preference was given to studies providing mechanistic interpretation, strain-level validation, or matrix-specific fermentation outcomes. Literature screening and study selection were performed primarily by the first author and critically evaluated through iterative discussion among co-authors to ensure conceptual consistency and minimize selection bias.

Study selection prioritized publications reporting strain-specific functionality, matrix-associated effects, and mechanistic interpretation of fermentation performance in brewing, wine, fruit, cereal, bakery, plant-based, and functional fermentation systems. Particular attention was devoted to studies addressing key functional determinants of fermentation performance, including carbon metabolism, aroma biogenesis, acidification, enzymatic activity, microbial interactions, stress adaptation, and transformation of food-associated metabolites. Studies focused exclusively on taxonomy without functional interpretation or lacking clear technological relevance were considered of lower priority unless they provided important conceptual context.

Because substantial heterogeneity exists among food matrices, microbial strains, fermentation strategies, and analytical methodologies, the present review was not intended as a systematic review or quantitative meta-analysis. Instead, evidence was critically synthesized to identify recurring mechanistic patterns, conceptual limitations of taxonomy-centered interpretation, and emerging principles supporting a more predictive and application-oriented understanding of yeast functionality. This synthesis subsequently served as the conceptual basis for development of the proposed matrix–trait–function framework discussed throughout the review.

## 3. From Taxonomy to Functionality: Beyond Species-Based Classification

### 3.1. Traditional Taxonomy-Based Perspective

The study of non-*Saccharomyces* yeasts in food fermentation has historically developed through a taxonomy-centered perspective, in which microbial functionality is predominantly interpreted according to species identity and phylogenetic classification. This approach has provided an important foundation for understanding yeast biodiversity, ecological adaptation, and technological relevance across multiple fermentation systems. Consequently, numerous food-associated yeasts have been characterized according to their dominant metabolic or technological features, such as aroma enhancement (*Torulaspora delbrueckii, Pichia kluyveri*), acidification potential (*Lachancea thermotolerans*), reduced ethanol formation (*Metschnikowia pulcherrima, Cyberlindnera saturnus*), or bioprotection and microbial antagonism (*Metschnikowia pulcherrima, Wickerhamomyces anomalus*). Such classification has substantially contributed to the industrial adoption of non-*Saccharomyces* yeasts by simplifying species selection according to targeted fermentation objectives [2,5,6].

However, taxonomy-driven interpretation inherently assumes a degree of functional consistency within species that may not always reflect biological reality. Many currently used descriptors, including “aroma-producing yeast”, “low-alcohol yeast”, or “acidifying yeast”, implicitly generalize functionality at the species level, despite growing evidence that fermentation-relevant phenotypes are frequently strain-specific and strongly dependent on environmental conditions. As a consequence, species identity alone often provides limited predictive power regarding actual fermentation performance, particularly when yeasts are transferred across different food matrices or processing environments [3,22].

### 3.2. Why Taxonomy Alone Is Often Insufficient: Strain Specificity and Functional Variability

Although taxonomy-based classification provides a useful framework for organizing yeast diversity, increasing evidence suggests that fermentation-relevant functionality cannot be reliably inferred from species identity alone. Considerable phenotypic heterogeneity has been reported among strains belonging to the same species, particularly regarding sugar utilization patterns, aroma production, organic acid metabolism, stress tolerance, enzymatic activity, and microbial interactions [3,5]. Consequently, strains classified within a single taxonomic group may exhibit substantially different technological behavior under comparable fermentation conditions.

For example, species frequently associated with reduced ethanol formation, including *Metschnikowia pulcherrima* or *Cyberlindnera saturnus*, do not consistently display identical fermentative performance across studies [5,23], while strains of *Torulaspora delbrueckii* and *Pichia kluyveri* may generate markedly different volatile profiles depending on strain background [24,25]. Likewise, the acidification capacity of *Lachancea thermotolerans* frequently differs across strains, suggesting that broad species-level descriptors may oversimplify microbial functionality and reduce predictive applicability in food fermentation systems [7,8].

Importantly, strain-level functional divergence likely reflects underlying variability in metabolic regulation, stress adaptation, nutrient utilization, enzymatic expression, and ecological competitiveness. Consequently, fermentation-relevant functionality should increasingly be interpreted as a strain-associated property rather than as a universally transferable species characteristic [22,26].

As a result, taxonomy-centered terminology such as “aroma-producing yeast”, “acidifying yeast”, or “low-alcohol yeast” may provide only limited predictive value when applied without strain-level validation [2,3,6]. A functionality-oriented interpretation, therefore, requires moving beyond species designation toward the evaluation of microbial traits directly associated with targeted technological outcomes [9,10,22].

### 3.3. Functional Redundancy and Functional Divergence Among Non-Saccharomyces Yeasts

A further limitation of taxonomy-centered interpretation is that fermentation functionality does not always follow a one-species–one-function logic. Different non-*Saccharomyces* species may contribute to similar technological outcomes through distinct metabolic routes, while strains belonging to the same species may display divergent functional behavior depending on their genotype, physiological state, and fermentation environment [3,5]. This creates both functional redundancy, whereby multiple yeasts may contribute to comparable fermentation outcomes, and functional divergence, in which taxonomically related strains display substantially different technological performance [12,24].

Functional redundancy is particularly relevant in product design because similar fermentation objectives can be achieved using different microbial strategies. For instance, reduced ethanol formation may result from limited maltose or maltotriose utilization, preferential respiration under oxygenated conditions, restricted fermentative capacity, or interactions with *S. cerevisiae* in sequential or mixed fermentations [2,6,8]. Similarly, aroma enhancement may arise through ester formation, higher alcohol metabolism, thiol release, terpene liberation, or glycosidase-mediated release of aroma precursors, depending on the yeast strain and substrate composition [2,7,26].

Conversely, functional divergence explains why yeasts assigned to the same species cannot necessarily be treated as interchangeable technological units. Strain-level differences in sugar utilization, nitrogen metabolism, stress tolerance, enzymatic activity, and volatile compound formation may result in distinct fermentation kinetics and product profiles even when strains are applied to the same substrate [5,13]. This distinction is particularly relevant for industrial translation, where reproducibility and process predictability frequently require strain-level characterization rather than reliance on species identity alone [12].

From an application perspective, these concepts have important implications for starter culture selection. If taxonomically distinct yeasts can achieve comparable technological outcomes, selection should be guided by compatibility among target product characteristics, matrix constraints, safety status, process robustness, and sensory objectives rather than taxonomy alone [8,10]. Conversely, when strains within the same species exhibit substantial functional divergence, species-level claims should be interpreted cautiously and supported by strain-specific validation under relevant fermentation conditions [3,24]. A functionality-oriented perspective, therefore, does not replace taxonomy but rather complements it by emphasizing the traits that determine fermentation outcomes within specific matrix and process contexts [27].

### 3.4. Toward a Functionality-Oriented Classification of Yeast Diversity

The increasing recognition of strain-level variability highlights the need to complement taxonomy-driven classification with a more functionality-oriented perspective. While taxonomic identification remains essential for understanding phylogeny, ecology, safety assessment, and regulatory considerations, its predictive value for fermentation performance may remain limited when applied in isolation [3,24]. In practical food fermentation systems, technological outcomes are rarely determined by species identity alone but instead emerge from interactions among microbial metabolic potential, food matrix composition, and process conditions.

From an applied perspective, a functionality-oriented classification may offer greater relevance for fermentation design than taxonomy-centered descriptions alone. Rather than selecting yeasts primarily according to species designation, targeted microbial selection may increasingly benefit from evaluation of key metabolic and technological traits associated with desired fermentation outcomes. Depending on the application, these may include carbon metabolism, aroma biogenesis, acidification potential, enzymatic functionality, microbial competitiveness, stress tolerance, and metabolite transformation capacity [2,6].

Importantly, the technological relevance of these traits remains context-dependent. Functional characteristics that are advantageous in one fermentation system may become undesirable in another depending on substrate composition, environmental conditions, and product objectives. Consequently, microbial functionality should be interpreted not as a fixed species-associated property but rather as an emergent feature shaped by interactions between microbial traits and the fermentation environment.

This perspective provides the conceptual basis for the matrix–trait–function framework proposed in the present review and highlights the importance of understanding how food matrix composition influences microbial performance. The following section, therefore, focuses specifically on the role of food matrix characteristics as determinants of yeast functionality, while representative examples of dominant functional traits among selected food-associated non-*Saccharomyces* yeasts are summarized in Table 1.

## 4. Food Matrix as a Determinant of Yeast Functionality

While strain specificity represents an important determinant of microbial performance, fermentation outcomes are additionally shaped by interactions between microbial metabolism and the physicochemical properties of the surrounding food matrix. Rather than functioning as passive substrates, food matrices actively influence microbial behavior by modulating nutrient availability, metabolic regulation, enzymatic functionality, microbial competitiveness, and stress adaptation [14,15,16,17].

Importantly, matrix effects extend beyond simple compositional differences and frequently determine whether strain-associated metabolic potential can be effectively expressed under specific fermentation conditions. Factors including sugar composition, nitrogen availability, oxygen transfer, pH, precursor abundance, polyphenol composition, osmotic pressure, and indigenous microbial ecology may substantially influence aroma formation, ethanol production, acidification, enzymatic activity, and microbial interactions [15,17,37].

Consequently, microbial functionality should be interpreted as context-dependent rather than universally transferable across fermentation systems. Functional traits considered advantageous in one substrate may become technologically irrelevant or even undesirable in another depending on fermentation objectives and environmental constraints. The following sections, therefore, examine how food matrix composition modulates microbial functionality across representative fermentation systems.

### 4.1. Brewing Systems: Matrix Constraints and Functional Opportunities

Brewing systems represent a highly structured fermentation environment in which yeast functionality is strongly shaped by wort composition, hop-derived compounds, fermentation temperature, oxygen availability, and process design. Unlike fruit-based fermentations dominated by readily fermentable sugars, brewing substrates contain high proportions of maltose and maltotriose, limited simple sugars, restricted oxygen availability after pitching, and antimicrobial hop metabolites, creating conditions that strongly influence sugar utilization, ethanol production, aroma formation, and microbial competitiveness [2,4,6].

Carbon source composition represents one of the principal brewing-specific determinants of yeast functionality. Because wort carbohydrates are dominated by maltose and maltotriose, yeasts lacking efficient transport and utilization systems for these sugars frequently exhibit restricted fermentative performance and reduced ethanol production.

While this characteristic may represent a limitation in conventional brewing, it has attracted increasing technological interest for the production of low- and non-alcoholic beers, where restricted sugar metabolism may become advantageous. Species including *Metschnikowia pulcherrima*, *Cyberlindnera saturnus*, *Pichia kluyveri*, and selected strains of *Torulaspora delbrueckii* have therefore been investigated for controlled ethanol modulation combined with aroma enhancement [3,8,22]. However, technological performance remains highly dependent on wort composition, fermentation strategy, and strain-associated metabolic capacity, particularly in co-fermentation or sequential inoculation systems with *Saccharomyces cerevisiae* [5].

Hop-derived compounds constitute another brewing-specific determinant influencing microbial functionality. Iso-α-acids and related hop metabolites may affect microbial competitiveness and stress adaptation, while glycosidically bound aroma precursors and terpenes may interact with yeast enzymatic systems, particularly β-glucosidases, thereby influencing aroma liberation and sensory complexity [9,31]. These interactions illustrate how brewing matrices may not only constrain microbial functionality but also create opportunities for targeted modulation of flavor-active compounds through strain-specific enzymatic activity.

Nitrogen availability further represents an important brewing determinant shaping yeast metabolism. Free amino nitrogen (FAN) regulates biomass formation, carbon flux redistribution, redox homeostasis, and volatile compound production, particularly through amino acid catabolism associated with higher alcohol and ester formation [26,38,39]. Consequently, variability in wort nitrogen composition may substantially reshape aroma development and fermentation performance, even among strains exhibiting otherwise similar fermentative characteristics [30,37,40].

Collectively, brewing systems illustrate how fermentation functionality emerges through interactions between matrix composition and microbial metabolic potential rather than through species identity alone [41,42]. The brewing environment, therefore, represents an informative model system for understanding how matrix-specific constraints shape the technological performance of non-*Saccharomyces* yeasts.

### 4.2. Wine and Fruit Fermentations: Sugar-Rich Environments and Aroma-Driven Functionality

Wine and fruit fermentations represent physicochemically distinct environments from brewing systems and therefore impose fundamentally different constraints on yeast metabolism. Unlike wort-based fermentations dominated by maltose and maltotriose, grape musts and fruit substrates are typically characterized by elevated concentrations of readily fermentable sugars, predominantly glucose and fructose, acidic pH, increased osmotic pressure, diverse phenolic composition, and abundant glycosylated aroma precursors [6,10,15]. These matrix-specific conditions strongly influence carbon metabolism, stress adaptation, volatile compound biosynthesis, and enzymatic activity, frequently favoring sensory diversification and aroma complexity rather than ethanol restriction alone.

One of the principal functional consequences of fruit- and grape-derived matrices lies in their high abundance of aroma precursors. Non-*Saccharomyces* yeasts increasingly attract attention for their capacity to modulate volatile composition through ester formation, higher alcohol production, terpene release, volatile thiol formation, and enzymatic liberation of matrix-bound aroma compounds. Enzymatic activities, including β-glucosidase, esterase, and carbon–sulfur lyase activity, may facilitate the release of glycosidically bound volatile precursors naturally present in grape and fruit substrates, thereby substantially influencing aroma complexity and sensory perception. Consequently, species including *Torulaspora delbrueckii*, *Metschnikowia pulcherrima*, *Pichia kluyveri*, *Hanseniaspora uvarum*, and *Lachancea thermotolerans* have attracted growing interest for targeted modulation of aroma complexity, acidity, and mouthfeel in wine production [32,43,44]. However, fermentation outcomes frequently remain highly dependent on strain selection, grape variety, nutrient composition, fermentation temperature, and co-fermentation strategies.

Organic acid composition represents another important determinant shaping microbial performance in wine and fruit fermentations. Low pH together with elevated concentrations of tartaric, malic, citric, and other organic acids may substantially influence stress adaptation, redox homeostasis, and microbial competitiveness. Under these conditions, certain non-*Saccharomyces* yeasts have attracted attention due to their capacity to modulate acidity and sensory balance through organic acid metabolism. For example, *Lachancea thermotolerans* has been extensively investigated for biological acidification through lactic acid production, particularly in warm-climate viticulture and low-acidity musts. Nevertheless, reported acidification performance frequently varies across strains and fermentation conditions, emphasizing the importance of matrix-dependent expression of microbial functionality [23,45,46].

Beyond aroma modulation, fruit matrices may additionally provide favorable conditions for yeast-mediated transformation of plant-derived metabolites. Elevated concentrations of phenolic compounds, glycosides, terpenes, and other aroma precursors create opportunities for enzymatic modification and release of bioactive or sensory-relevant compounds during fermentation. Although current evidence remains fragmented and frequently matrix-specific, fruit fermentations increasingly represent valuable systems for investigating microbial biotransformation processes extending beyond conventional ethanol production [47,48,49].

Compared with brewing systems, where matrix constraints frequently limit sugar utilization and fermentation efficiency, wine and fruit fermentations more commonly enable expression of aroma-related metabolic potential. Together, these observations further illustrate how matrix composition fundamentally shapes technological functionality of non-*Saccharomyces* yeasts across food fermentation systems.

### 4.3. Cereal and Bakery Systems: Nutrient Availability and Microbial Interactions

Cereal- and bakery-based fermentations constitute distinct microbial environments characterized by complex carbohydrate matrices, reduced concentrations of readily fermentable sugars, variable nutrient accessibility, and pronounced microbial interactions. Unlike brewing and fruit fermentations, cereal systems are predominantly structured around starch-derived substrates whose fermentability frequently depends on enzymatic hydrolysis and substrate accessibility [50,51,52]. Consequently, microbial functionality in these systems is strongly shaped by carbohydrate availability, fermentation duration, physicochemical conditions, and ecological interactions occurring within mixed microbial communities.

One of the defining characteristics of cereal fermentations is the close association between yeasts and lactic acid bacteria (LAB), particularly in sourdough systems. Rather than functioning independently, non-*Saccharomyces* yeasts frequently coexist with LAB in dynamic microbial consortia where nutrient competition, cross-feeding, metabolite exchange, and environmental modification collectively shape fermentation trajectories [53,54,55]. Organic acid production by LAB substantially alters pH and microbial selection pressure, while yeast metabolism contributes to carbon dioxide generation, aroma formation, osmotic adaptation, and dough rheology. Consequently, microbial performance in cereal fermentations frequently reflects ecosystem-level interactions rather than isolated strain-associated traits alone.

From a technological perspective, cereal fermentations frequently favor traits associated with enzymatic functionality, stress tolerance, and sensory development. Enzymes involved in carbohydrate hydrolysis, proteolysis, and glycoside transformation may substantially influence dough structure, nutrient accessibility, flavor generation, and texture-related properties of fermented cereal products. In addition, selected non-*Saccharomyces* yeasts have increasingly attracted attention for improving aroma complexity, shelf stability, and nutritional quality through interactions with endogenous cereal metabolites and microbial consortia [30,56,57].

An additional source of functional variability arises from heterogeneity of cereal substrates themselves. Differences in starch composition, fiber content, protein structure, mineral availability, and endogenous enzymatic activity may substantially alter microbial adaptation and fermentation performance [58,59,60]. Compared with brewing and wine systems, where fermentation outcomes are often driven primarily by sugar metabolism or aroma precursor availability, cereal fermentations more strongly emphasize ecological interactions and substrate accessibility as determinants of microbial functionality. These systems therefore further illustrate how fermentation performance emerges through interactions between microbial metabolism, matrix composition, and surrounding microbial ecology.

### 4.4. Plant-Based Fermented Foods and Functional Beverages: Matrix Complexity and Emerging Functional Opportunities

Plant-based fermented foods and functional beverages represent highly heterogeneous fermentation systems characterized by diverse carbohydrate profiles, elevated concentrations of phenolic compounds, glycosylated metabolites, organic acids, and structurally complex plant-derived bioactive compounds [1,61,62]. Compared with brewing or cereal fermentations, these matrices frequently exhibit greater variability in nutrient accessibility, precursor abundance, and phytochemical composition, thereby creating distinct metabolic environments for microbial activity. Consequently, functionality of non-*Saccharomyces* yeasts in plant-derived systems frequently extends beyond conventional ethanol production and aroma modulation toward broader interactions with matrix-associated compounds.

One of the defining characteristics of plant-derived matrices is the abundance of glycosylated aroma precursors, phenolic compounds, terpenes, flavonoids, and matrix-bound metabolites whose accessibility may change during fermentation. In this context, enzymatic systems associated with selected non-*Saccharomyces* yeasts, including β-glucosidases, esterases, and other hydrolytic enzymes, have attracted increasing attention due to their potential to release or transform compounds contributing to aroma complexity, sensory diversification, and functional properties. However, the magnitude and direction of these transformations frequently depend on precursor availability, matrix composition, fermentation conditions, and strain-associated metabolic activity, limiting broad transferability across food systems [47,48,49].

The growing demand for functional beverages and low- or non-alcoholic fermented products has further expanded interest in exploiting yeast diversity for targeted technological functionality. In such systems, fermentation objectives frequently extend beyond ethanol production and increasingly involve modulation of sensory properties, enhancement of microbial stability, reduction of undesirable compounds, or altered accessibility of selected food-associated metabolites. These objectives have stimulated growing interest in mixed fermentations and non-conventional yeast systems capable of balancing technological robustness with desired functional outcomes [13,44,63]. Nevertheless, predictive understanding of matrix-dependent metabolic transformations remains limited, particularly regarding reproducibility across substrates and fermentation settings.

Importantly, plant-derived matrices increasingly provide valuable experimental systems for investigating yeast-mediated transformation of food-associated bioactive compounds, including phenolics, glycosides, terpenes, and other secondary metabolites. Although current evidence remains fragmented and often highly product-specific, these fermentations increasingly emphasize the broader role of non-*Saccharomyces* yeasts as modulators of matrix functionality rather than merely fermentative organisms [64,65,66]. This emerging perspective further provides an important conceptual bridge toward the microbial transformation processes discussed later in this review.

Collectively, the examples discussed above demonstrate that fermentation performance emerges through interactions among food matrix characteristics, strain-associated metabolic capabilities, and process conditions. Similar microbial traits may therefore generate substantially different technological outcomes depending on matrix composition, ecological context, and fermentation objectives. Representative matrix–trait–function relationships across major food fermentation systems are summarized in Table 2.

**Figure 2:**
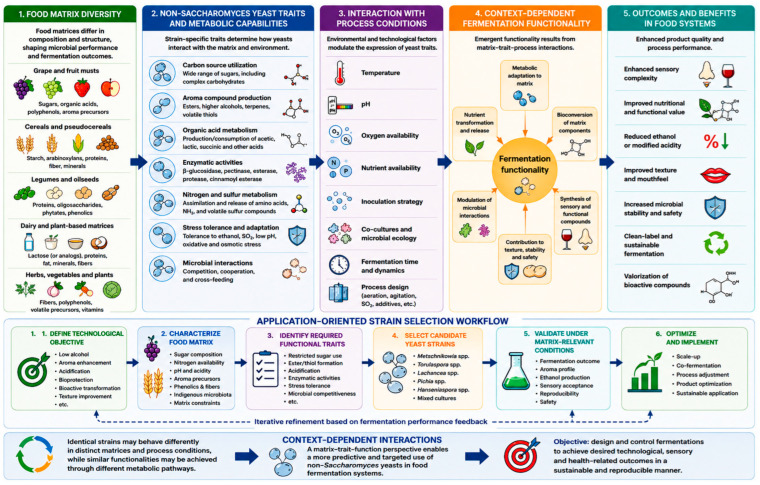
Matrix–trait–function interactions governing non-*Saccharomyces* yeast performance across food fermentation systems. The schematic summarizes key interactions among food matrix characteristics, strain-specific microbial traits, and process conditions that collectively shape fermentation functionality and technological outcomes. The framework highlights the context-dependent nature of yeast performance, whereby similar microbial traits may generate distinct outcomes depending on substrate composition, precursor availability, microbial ecology, and environmental constraints. By integrating these interactions across major food fermentation systems, the framework provides a more predictive and application-oriented perspective for targeted yeast selection and fermentation design.

Taken together, the evidence discussed above demonstrates that fermentation performance emerges through dynamic interactions among food matrix composition, strain-specific microbial capabilities, and process conditions. Consequently, similar microbial traits may lead to distinct technological outcomes depending on matrix-specific constraints, ecological interactions, and environmental parameters. Across food fermentation systems, matrix effects consistently shape microbial functionality through modulation of nutrient accessibility, precursor abundance, stress exposure, and microbial interactions, although the dominant drivers differ substantially among substrates. A practical overview of representative matrix–trait–function interactions governing non-*Saccharomyces* yeast performance across major food fermentation systems is provided in Figure 2.

## 5. Functional Metabolic Traits Driving Food Applications

Because fermentation outcomes emerge through interactions among microbial metabolism, food matrix composition, and process conditions, targeted yeast selection increasingly depends on understanding specific functional traits associated with desired technological objectives. Rather than representing fixed species-associated properties, these traits frequently display substantial strain-level variability and context dependency, emphasizing the importance of functionality-oriented interpretation for predictive fermentation design [12,13,24,44].

Among the principal determinants of fermentation performance are carbon metabolism, aroma biosynthesis, acidification potential, enzymatic activity, microbial competitiveness, and stress tolerance. However, the technological relevance of individual traits varies considerably across food systems depending on matrix composition, precursor availability, fermentation objectives, and process constraints. For example, restricted sugar utilization may become advantageous in low- and non-alcoholic brewing, whereas extensive fermentative performance often remains desirable in wine or cereal fermentations. Similarly, enzymatic activities contributing to aroma liberation or metabolite transformation may play major roles in plant-derived systems yet remain less relevant in matrices lacking suitable precursor compounds. Consequently, microbial functionality is best interpreted through a matrix–trait–function perspective, in which metabolic traits acquire technological relevance only within specific fermentation contexts [30,54,55,66].

### 5.1. Carbon Metabolism and Ethanol Modulation

Carbon metabolism represents one of the principal determinants of fermentation functionality because it governs substrate utilization, ethanol formation, biomass generation, redox balancing, and the synthesis of fermentation-derived metabolites [3]. In non-*Saccharomyces* yeasts, considerable diversity has been reported in carbohydrate transport, fermentative efficiency, respiratory metabolism, and carbon flux regulation, contributing to pronounced variability in technological performance across strains and fermentation systems [12,24].

Substrate utilization represents one of the primary mechanisms underlying functional divergence among non-*Saccharomyces* yeasts. While many strains efficiently metabolize glucose and fructose, utilization of disaccharides and trisaccharides such as maltose and maltotriose frequently remains limited or strain-dependent [5,38]. This restricted carbohydrate metabolism may substantially alter fermentation performance by limiting ethanol accumulation and modifying residual sugar composition, a characteristic increasingly exploited in low- and non-alcoholic beverage production where incomplete sugar conversion may become technologically advantageous [8,22].

Species including *Metschnikowia pulcherrima*, *Cyberlindnera saturnus*, *Pichia kluyveri*, and selected strains of *Torulaspora delbrueckii* have therefore attracted interest for controlled ethanol modulation in brewing and beverage fermentations [33]. However, reported outcomes frequently vary across studies, indicating that ethanol reduction reflects interactions among strain-associated metabolic capacity, oxygen availability, nutrient composition, and process design rather than a fixed species-associated property [5,26].

Beyond substrate uptake, fermentation performance is further shaped by intracellular carbon partitioning. Variability in Crabtree behavior, respiratory metabolism, glucose repression, and mitochondrial activity may redirect carbon flux toward ethanol, biomass, glycerol, organic acids, or volatile metabolites [24,67]. Under oxygenated or nutrient-variable conditions, some non-*Saccharomyces* yeasts may partially favor respiratory metabolism, thereby reducing ethanol accumulation while simultaneously influencing aroma formation and redox homeostasis [68,69].

Carbon metabolism additionally interacts with osmotic adaptation and intracellular redox balancing. Increased glycerol production, frequently observed in selected non-*Saccharomyces* yeasts, may facilitate NADH reoxidation under stress-associated conditions while simultaneously contributing to mouthfeel and sensory perception [40,69]. Changes in carbon allocation may further influence organic acid and volatile compound formation, illustrating that ethanol modulation rarely emerges through a single metabolic mechanism but instead reflects trade-offs among energy generation, redox balancing, microbial growth, and stress adaptation [70,71].

The technological significance of carbon metabolism ultimately depends on fermentation objectives and matrix-specific constraints. Restricted fermentative capacity may represent a limitation in systems requiring complete sugar utilization while becoming advantageous in products targeting ethanol reduction or altered sensory balance [10,72]. Consequently, carbon metabolism is best interpreted as a functionality-oriented trait emerging through interactions among substrate preference, metabolic regulation, and fermentation objectives rather than through taxonomy-based assumptions alone [3].

### 5.2. Aroma Biogenesis and Sensory Modulation

Aroma modulation represents one of the most extensively exploited functional traits of non-*Saccharomyces* yeasts because it directly contributes to sensory diversification and product differentiation in fermented foods and beverages. Unlike ethanol formation, which is primarily governed by central carbon metabolism, aroma biogenesis emerges through the coordinated interactions among multiple metabolic pathways involved in volatile synthesis, precursor transformation, and aroma release [3]. Consequently, substantial variability in sensory outcomes is frequently observed across strains and fermentation systems [6].

Volatile compound formation in non-*Saccharomyces* fermentations is primarily influenced by amino acid catabolism, ester biosynthesis, sulfur metabolism, lipid metabolism, and enzymatic release of aroma precursors [24]. Rather than emerging from isolated metabolic traits, aroma outcomes frequently reflect interactions among carbon allocation, nitrogen metabolism, precursor availability, and intracellular redox balancing [2].

Ester biosynthesis represents one of the principal mechanisms contributing to fruity and floral sensory perception. Ester formation is closely associated with alcohol acetyltransferase activity and depends on intracellular acetyl-CoA availability, precursor alcohol production, and cellular redox balance [72]. Consequently, differences in ester production frequently arise through broader variation in carbon metabolism and nitrogen assimilation rather than isolated enzymatic activity alone [26].

Higher alcohol formation is strongly linked to amino acid catabolism through the Ehrlich pathway, which converts amino acids into fusel alcohols and related aroma-active metabolites [5]. Nitrogen availability, amino acid composition, and intracellular nitrogen metabolism may therefore substantially influence sensory profiles by altering precursor fluxes and redox-associated metabolic responses during fermentation [39].

Sulfur-containing volatile compounds represent another important dimension of aroma modulation. Selected non-*Saccharomyces* yeasts may contribute to volatile thiol release through carbon–sulfur lyase activity, particularly in substrates rich in glycosylated or cysteinylated aroma precursors [30]. In addition, microbial metabolism may indirectly reshape sulfur-associated aroma formation by modifying precursor accessibility, redox homeostasis, and microbial interactions during fermentation [29]. However, sulfur-associated sensory outcomes frequently remain highly dependent on precursor abundance, oxygen exposure, fermentation conditions, and microbial interactions, contributing to substantial variability across strains and fermentation systems [32,41].

Beyond de novo volatile synthesis, aroma modulation may additionally emerge through enzymatic transformation of naturally occurring non-volatile precursors. β-Glucosidase and esterase activities have frequently been associated with liberation of terpenes, glycosidically bound volatiles, and other aroma-active compounds, particularly in fruit-, grape-, botanical-, and hop-derived substrates [18,31]. Nevertheless, the technological significance of these activities frequently depends on fermentation conditions because glucose repression, ethanol accumulation, pH, precursor abundance, and oxygen availability may substantially influence enzyme expression and aroma release efficiency [21,51].

Species including *Torulaspora delbrueckii*, *Pichia kluyveri*, *Metschnikowia pulcherrima*, *Hanseniaspora uvarum*, and selected strains of *Lachancea thermotolerans* have repeatedly attracted interest for aroma modulation in fermented foods and beverages [23]. However, sensory outcomes frequently differ among studies because fermentation temperature, nitrogen composition, inoculation timing, oxygen transfer, and co-fermentation strategies may substantially reshape volatile compound production, even within the same species [16,73]. Consequently, aroma enhancement observed under one fermentation setting may not necessarily translate into reproducible outcomes elsewhere [7].

Aroma biogenesis should therefore not be interpreted through simplified descriptors such as “aroma-producing yeast.” Similar sensory outcomes may emerge through distinct biochemical routes, while comparable metabolic traits may generate divergent sensory consequences depending on fermentation objectives, precursor availability, and process conditions. Aroma modulation is thus best understood as a multidimensional functional trait emerging from interactions among microbial metabolism, food matrix composition, and fermentation design rather than as a fixed species-associated property [3,10,24].

### 5.3. Acidification, Mouthfeel, and Texture-Related Functionality

Beyond ethanol production and aroma modulation, non-*Saccharomyces* yeasts may substantially influence sensory perception through metabolic activities affecting acidity, mouthfeel, texture, and structural properties of fermented foods and beverages. These functionalities frequently emerge through interconnected mechanisms involving organic acid production, glycerol formation, mannoprotein release, extracellular polysaccharide production, and interactions with food macromolecules [3]. Consequently, sensory outcomes often arise through coordinated metabolic responses rather than isolated technological traits [6].

Organic acid metabolism represents one of the most important mechanisms through which non-*Saccharomyces* yeasts may modulate sensory balance and microbial stability. Changes in concentrations of lactic, succinic, acetic, malic, and citric acids may substantially alter perceived freshness, acidity, flavor balance, and fermentation dynamics [16,26]. Because organic acid metabolism is closely associated with intracellular redox balancing and carbon allocation, acidification may additionally influence microbial competitiveness and process robustness [24].

Among non-*Saccharomyces* yeasts, *Lachancea thermotolerans* has attracted particular attention because of its capacity for biological acidification through lactic acid production [28]. This functionality has increasingly been explored in wine and brewing systems, particularly where acidity adjustment may improve sensory freshness or microbial stability [23]. Nevertheless, acidification outcomes frequently vary across strains and fermentation conditions, including temperature, nutrient composition, oxygen availability, substrate chemistry, and inoculation strategy [39,74]. Consequently, acidification should not be interpreted as a universally predictable or inherently beneficial technological trait.

Mouthfeel-related functionality frequently arises through glycerol production and associated redox balancing. Because glycerol synthesis contributes to NADH reoxidation and osmotic stress adaptation, several non-*Saccharomyces* yeasts may exhibit elevated glycerol formation compared with conventional fermentative systems [24,40]. Increased glycerol concentrations may contribute to enhanced viscosity, sweetness perception, body, and overall sensory fullness, particularly in low-ethanol products where reduced alcohol content may otherwise diminish mouthfeel [69]. However, glycerol-associated sensory effects remain concentration-dependent and may interact with ethanol content, residual sugars, acidity, and aroma compounds, frequently resulting in complex sensory trade-offs [71].

Texture-related functionality may additionally arise through yeast-derived mannoproteins and extracellular polysaccharides released during fermentation or autolysis. These compounds may interact with proteins, polyphenols, colloidal structures, and starch-derived polymers, potentially influencing viscosity, foam stability, dough rheology, colloidal properties, and overall product texture [46,59]. In fermented beverages, mannoproteins may further influence aroma retention and colloidal stability through interactions with volatile compounds and phenolic constituents [56,57]. Consequently, texture-related functionality frequently overlaps with broader sensory outcomes rather than operating as an isolated technological feature.

Importantly, acidification, glycerol production, and texture modification remain metabolically interconnected because they emerge through central carbon metabolism, stress adaptation, and intracellular redox balancing [24]. Changes in one metabolic trait may therefore indirectly reshape other physicochemical and sensory properties of fermented products, reinforcing the systems-level nature of microbial functionality [70].

Accordingly, mouthfeel- and texture-related functionality should not be interpreted through simplified descriptors such as “body-enhancing yeast” or “acidifying yeast.” Their technological significance depends on balancing metabolic outcomes with sensory acceptability, matrix composition, and product-specific objectives [3,10].

### 5.4. Bioprotection and Microbial Interactions

Bioprotection represents an increasingly relevant functional trait of non-*Saccharomyces* yeasts, particularly in fermentation systems where microbial stability, spoilage control, and reduction of chemical preservatives constitute important technological objectives [3]. Unlike conventional antimicrobial interventions, yeast-mediated bioprotection frequently emerges through ecological interactions involving nutrient competition, environmental modification, secretion of inhibitory compounds, and interference with competing microorganisms [7].

Competition for nutrients represents one of the principal mechanisms shaping microbial interactions during fermentation. Rapid uptake of fermentable substrates, amino acids, oxygen, vitamins, and trace elements by selected non-*Saccharomyces* yeasts may suppress spoilage organisms and alter microbial succession patterns during fermentation [75]. Such competitive interactions may substantially influence fermentation trajectories even in the absence of direct antimicrobial activity, emphasizing the ecological nature of microbial functionality [76].

Iron competition has attracted particular attention in selected yeast species, especially *Metschnikowia pulcherrima*, which produces pulcherriminic acid capable of chelating environmental iron and thereby restricting growth of competing microorganisms [31]. This mechanism has increasingly been investigated as a strategy for microbial stabilization in food and beverage fermentations, particularly where microbial competition strongly influences product quality and fermentation reproducibility [29].

Selected non-*Saccharomyces* yeasts may additionally produce killer toxins or other inhibitory metabolites capable of suppressing spoilage yeasts and undesirable microbial populations [34]. Species including *Wickerhamomyces anomalus* and selected strains of *Pichia* spp. have therefore attracted interest because of their antagonistic potential in food fermentation systems [36]. Nevertheless, antimicrobial effectiveness frequently varies according to pH, nutrient composition, microbial load, and fermentation conditions [35].

Microbial interactions may also emerge indirectly through environmental modification. Consumption of dissolved oxygen, shifts in pH, changes in nutrient availability, and alterations in metabolite composition may substantially reshape microbial ecology and fermentation dynamics, thereby influencing both beneficial and undesirable microorganisms [77]. These ecological changes may further affect microbial succession and fermentation reproducibility, particularly in mixed-culture fermentations [53].

In mixed fermentations, interactions may become particularly complex because non-*Saccharomyces* yeasts may either complement or compete with *Saccharomyces cerevisiae*. Sequential and co-inoculated fermentations may improve aroma complexity, contribute to ethanol modulation, and enhance microbial stability, yet may also result in delayed fermentation, incomplete attenuation, or unintended metabolite production depending on inoculation timing and ecological compatibility [29,78]. Microbial interactions should therefore be interpreted as dynamic ecological processes rather than fixed interspecies relationships [79].

The technological significance of microbial interactions extends beyond spoilage prevention and includes broader consequences for fermentation reproducibility, product quality, and process robustness [69]. However, microbial competitiveness should not automatically be interpreted as beneficial because excessive antagonism may negatively affect desirable starter cultures or disrupt intended fermentation trajectories [58].

Accordingly, predictive implementation of bioprotective functionality requires evaluation of microbial performance under fermentation-relevant conditions rather than reliance on broad species-associated assumptions alone [2,3].

### 5.5. Enzymatic Functionality and Metabolite Transformation

Enzymatic functionality represents an important mechanism through which non-*Saccharomyces* yeasts may influence fermentation outcomes beyond primary sugar conversion. Unlike metabolic traits primarily associated with carbon utilization or aroma synthesis, enzymatic activities may modify compounds that are initially structurally bound, non-volatile, or metabolically inaccessible within food matrices [3]. Consequently, enzymatic functionality may substantially influence substrate accessibility, volatile composition, nutrient release, and metabolite transformation across diverse fermentation systems [24].

Among enzymatic traits, β-glucosidase activity has received particular attention because of its capacity to hydrolyze glycosidically bound compounds and release aroma-active metabolites [31]. In grape-, fruit-, botanical-, and hop-derived fermentations, β-glucosidase activity may contribute to the liberation of terpenes and other volatile compounds that remain inaccessible in conjugated forms prior to fermentation [18]. Consequently, relatively subtle enzymatic modifications may substantially reshape aroma perception despite relatively limited changes in bulk metabolite composition.

The technological relevance of β-glucosidase activity nevertheless remains strongly dependent on fermentation context. Glucose repression, ethanol accumulation, pH, temperature, oxygen availability, and substrate composition may substantially influence enzyme expression and catalytic efficiency [21]. As a result, enzymatic potential reported under laboratory conditions may not necessarily translate into reproducible fermentation outcomes under industrially relevant settings [51].

Beyond β-glucosidases, non-*Saccharomyces* yeasts may express esterases, proteases, pectinases, cellulases, and other hydrolytic enzymes capable of modifying substrate structure and metabolite accessibility [2,80]. Such activities may influence aroma equilibria, nutrient release, polysaccharide restructuring, and mobilization of compounds embedded within plant-derived matrices [80]. Consequently, enzymatic functionality frequently extends beyond flavor modulation toward broader restructuring of food matrix composition.

In cereal and bakery fermentations, enzymatic activities may influence carbohydrate assesibility, dough rheology, and texture formation through interactions with starch-derived and proteinaceous substrates [10]. Similarly, in plant-based fermentations, hydrolytic enzymes may contribute to modification of fiber-associated compounds and broader compositional restructuring during fermentation [59]. These observations further demonstrate that enzymatic effects frequently emerge through interactions between microbial metabolism and substrate characteristics rather than through universal microbial properties.

Increasing evidence additionally suggests that selection non-*Saccharomyces* yeasts may contribute to transformation of food-associated metabolites, including glycosides, phenolic compounds, terpenes, and matrix-bound constituents [18,31]. Such modifications may involve hydrolysis, oxidation–reduction reactions, ester cleavage, or indirect release through matrix disruption during fermentation, thereby influencing both sensory and compositional outcomes.

However, interpretation of metabolite transformation frequently remains challenging because observed compositional changes may emerge not only through direct microbial biotransformation, but also through matrix-driven release, fermentation-associated extraction effects, or altered analytical accessibility [70]. Mechanistic evidence therefore remains essential when attributing compositional changes to specific microbial activities, particularly in chemically complex food systems [21].

Importantly, enzymatic functionality should not be interpreted as a universal species-associated property because enzyme expression frequently differs among strains and may vary substantially depending on fermentation conditions [3]. The presence of enzymatic potential alone therefore does not guarantee measurable technological effects or predictable metabolite transformation [2].

Accordingly, enzymatic functionality may be more appropriately interpreted as a mechanistic bridge linking microbial metabolism with substrate transformation rather than as an isolated technological feature [24]. This perspective becomes particularly relevant for understanding emerging evidence regarding microbial modification of food-associated compounds, which remains strongly influenced by strain-specific functionality, substrate composition, and analytical interpretation [3].

## 6. Yeast-Mediated Transformation of Food-Associated Bioactive Compounds

### 6.1. Emerging Evidence Beyond Conventional Fermentation Outcomes

While Section 5 addressed enzymatic functionality as a broader determinant of fermentation performance, the following section specifically examines emerging evidence regarding yeast-mediated transformation of food-associated bioactive compounds together with the mechanistic uncertainties associated with their interpretation. Conventional applications of non-*Saccharomyces* yeasts have primarily focused on technological outcomes including ethanol modulation, aroma diversification, acidification, texture enhancement, and microbial stabilization. However, increasing evidence suggests that yeast functionality during fermentation may extend beyond these traditionally recognized effects and involve broader compositional modification of food-associated compounds [4,10,81].

Recent studies increasingly report compositional changes involving glycosides, phenolic compounds, terpenes, and matrix-associated metabolites following fermentation with selected non-*Saccharomyces* yeasts. These observations have stimulated growing interest in the possibility that microbial metabolism may contribute not only to fermentation performance but also to transformation of compounds that remain chemically inaccessible, structurally bound, or metabolically inactive prior to fermentation [8,82,83].

Nevertheless, the currently available evidence remains highly heterogeneous and frequently difficult to compare across studies. Reported compositional changes often vary substantially according to strain selection, substrate composition, fermentation conditions, inoculation strategy, and analytical methodology, complicating mechanistic interpretation and limiting broad generalization at the species level [9,25,84].

Several mechanisms have been proposed to explain fermentation-associated compositional shifts. These include enzymatic hydrolysis of glycosidically bound compounds, transformation of phenolic structures, terpene liberation, ester cleavage, oxidation–reduction reactions, and release of matrix-bound metabolites during microbial growth or substrate restructuring. However, the relative contribution of each mechanism frequently remains insufficiently resolved [75,85,86].

Importantly, observed compositional changes should not automatically be interpreted as evidence of direct microbial biotransformation. Fermentation-associated shifts may additionally emerge through altered extractability, matrix disruption, release of structurally bound compounds, adsorption to yeast biomass, or changes in analytical accessibility during fermentation [11,87,88].

Collectively, the emerging evidence suggests that yeast functionality may extend beyond conventional fermentation outcomes, yet mechanistic understanding remains fragmented and strongly dependent on interactions among strain-associated metabolism, matrix composition, and fermentation conditions. Consequently, interpretation of these transformations requires careful distinction between direct microbial metabolism and broader fermentation-associated compositional effects [1,29,50].

### 6.2. Mechanisms of Bioactive Transformation

#### 6.2.1. Glycoside Cleavage and Liberation of Conjugated Compounds

One of the most frequently discussed mechanisms underlying fermentation-associated compositional modification involves the enzymatic cleavage of glycosidically bound compounds. Many plant-derived food matrices contain metabolites in conjugated forms that remain chemically stable, non-volatile, or poorly accessible prior to fermentation. Enzymatic hydrolysis of glycosidic bonds may therefore alter the accessibility and physicochemical behavior of selected compounds during microbial processing [18,31,89].

β-Glucosidase-mediated hydrolysis has attracted particular attention because of its potential to release glycosidically bound precursors. This activity has been associated with the liberation of volatile terpenes, aroma-active metabolites, and other conjugated compounds in grape-, fruit-, botanical-, and cereal-derived fermentations [21,83,90,91].

Glycoside cleavage may additionally influence compounds extending beyond volatile aroma constituents. Certain flavonoids, phenolic glycosides, and matrix-associated metabolites may undergo partial liberation through hydrolytic enzyme activity, potentially reshaping compositional profiles during fermentation. However, reported outcomes frequently remain substrate-dependent and analytically heterogeneous [18,59,92].

Importantly, β-glucosidase activity itself frequently exhibits strong strain specificity and environmental sensitivity. Glucose repression, ethanol accumulation, oxygen exposure, pH, fermentation temperature, and precursor abundance may substantially influence enzymatic expression and catalytic efficiency. Consequently, enzymatic potential observed under controlled experimental conditions may not necessarily translate into reproducible compositional changes in industrial fermentation systems [29,51,93].

Interpretation of glycoside cleavage also requires methodological caution because observed increases in free compounds do not always indicate direct microbial transformation. Fermentation-associated matrix disruption, altered extractability, chemical hydrolysis, or changes in analytical accessibility may additionally contribute to compositional shifts reported after fermentation [77,87,88].

Therefore, glycoside cleavage may represent an important mechanism contributing to fermentation-associated compositional restructuring, although its technological significance remains strongly dependent on strain-specific enzymatic activity, substrate composition, and process conditions [1,3].

#### 6.2.2. Phenolic Transformation

Phenolic transformation has emerged as one of the most discussed yet mechanistically complex aspects of fermentation-associated compositional change in food systems. Increasing evidence suggests that selected non-*Saccharomyces* yeasts may influence phenolic composition during fermentation, although the extent and mechanisms of transformation frequently remain incompletely resolved [18,31,82].

Phenolic modifications during fermentation may arise through several overlapping mechanisms, including enzymatic hydrolysis, oxidation–reduction reactions, esterase-mediated cleavage, decarboxylation, adsorption to yeast cell walls, and indirect release from structurally bound matrix fractions. Consequently, observed compositional changes frequently reflect multiple simultaneous processes rather than a single transformation pathway [21,86,88].

Hydrolytic enzyme activities may contribute to cleavage of esterified or glycosylated phenolic structures, potentially increasing the accessibility of free phenolic compounds during fermentation. In plant-derived substrates, this process may facilitate partial liberation of phenolic constituents that remain structurally associated with polysaccharides, proteins, or fiber-rich matrix components prior to microbial processing [18,48,59,92].

Oxidation–reduction reactions may additionally reshape phenolic profiles through microbial metabolism or altered redox conditions during fermentation. Certain yeasts have been associated with modification of phenolic acids, flavonoid derivatives, and related metabolites, although mechanistic evidence frequently remains limited and highly strain-dependent [21,31,50,51].

Adsorption to yeast biomass represents another important yet frequently overlooked mechanism contributing to altered phenolic composition. Phenolic compounds may bind to mannoproteins, glucans, and other cell wall-associated structures, potentially reducing measurable concentrations in fermented products despite the absence of direct metabolic transformation [46,61,70].

Importantly, observed shifts in phenolic profiles should not automatically be interpreted as evidence of true microbial biotransformation. Apparent increases or decreases in phenolic concentrations may additionally reflect matrix disruption, fermentation-associated release of bound compounds, altered extractability, chemical instability, or differences in analytical accessibility before and after fermentation [57,70,77,87].

Interpretation becomes particularly challenging in complex food matrices where fermentation simultaneously alters pH, redox balance, enzymatic activity, microbial ecology, and substrate accessibility. As a result, direct attribution of phenolic changes to a single microbial mechanism frequently remains difficult without targeted metabolomic or mechanistic validation [24,68,94].

Current evidence therefore suggests that non-*Saccharomyces* yeasts may contribute to phenolic restructuring during fermentation, yet the biological significance and reproducibility of these transformations remain strongly context-dependent. Consequently, mechanistic interpretation should prioritize strain-specific validation and careful distinction between direct microbial metabolism and broader fermentation-associated compositional change [1,2,3].

#### 6.2.3. Terpene Liberation and Aroma-Associated Precursors

Terpene liberation represents one of the most frequently discussed mechanisms through which non-*Saccharomyces* yeasts may influence aroma-associated compositional changes during fermentation. In many plant-derived substrates, including grapes, fruits, botanicals, and hops, terpenes frequently occur in glycosidically bound or otherwise conjugated forms that exhibit limited volatility and reduced sensory accessibility prior to fermentation [18,31,82].

Enzymatic hydrolysis of glycosidically bound precursors may contribute to terpene liberation and release of volatile aroma-active compounds. This process has been associated with increased concentrations of monoterpenes and related compounds contributing to floral, citrus, and fruity sensory attributes in selected fermentation systems [83,90,91].

Beyond β-glucosidase-mediated hydrolysis, additional enzymatic processes including esterase activity and carbon–sulfur lyase-mediated reactions may further contribute to transformation of aroma-associated precursors and volatile release. Consequently, terpene-related aroma modulation frequently reflects coordinated enzymatic activity rather than a single metabolic pathway [29,30,69,95].

However, terpene liberation remains strongly dependent on substrate composition and precursor availability. Because many volatile compounds are initially present in low abundance or structurally inaccessible forms, the magnitude of fermentation-associated aroma modification may vary substantially across food matrices and fermentation systems [9,41,51].

Enzymatic efficiency may additionally be influenced by environmental factors including glucose repression, ethanol accumulation, pH, fermentation temperature, oxygen exposure, and microbial competition. Consequently, terpene liberation frequently remains difficult to predict solely from species identity or reported enzymatic potential [12,23,29,93].

Importantly, increased terpene concentrations observed after fermentation should not automatically be interpreted as direct evidence of microbial synthesis. In many cases, apparent increases may reflect liberation of pre-existing matrix-bound precursors, altered extractability, or shifts in volatile partitioning during fermentation rather than de novo metabolite production [18,57,70].

Current evidence therefore suggests that non-*Saccharomyces* yeasts may contribute to terpene-related aroma restructuring, although the reproducibility and technological relevance of these effects remain strongly context-dependent and require strain-level validation [1,2,3].

#### 6.2.4. Matrix-Bound Metabolites and Fermentation-Associated Release

During fermentation, microbial growth and enzymatic activity may contribute to restructuring of the surrounding matrix through degradation of cell wall components and hydrolysis of conjugated structures [21,51]. Fermentation may additionally modify polysaccharide organization and physicochemical conditions, including pH and redox balance, thereby influencing substrate accessibility and extractability [77,87]. Such changes may facilitate release of compounds that were previously matrix-bound or analytically inaccessible [57].

Beyond direct enzymatic transformation, increasing attention has been directed toward the possibility that fermentation may alter accessibility of compounds initially embedded within structurally complex food matrices. Many plant-derived substrates contain metabolites associated with polysaccharides, proteins, lipids, fiber structures, or cellular compartments, potentially limiting extractability and measurable availability prior to microbial processing [18]. In several plant-derived systems, matrix interactions have been shown to influence compound accessibility through structural association with cell wall components and insoluble fractions [59,96].

Fermentation-associated release has been discussed for multiple classes of compounds, including phenolics, glycosides, terpenes, peptides, amino acid derivatives, and other matrix-associated metabolites [18,31]. However, the relative contribution of microbial metabolism and matrix restructuring frequently remains difficult to distinguish experimentally, particularly in chemically complex food systems where multiple mechanisms may operate simultaneously [57,97].

Importantly, increased concentrations of detectable compounds following fermentation should not necessarily be interpreted as evidence of microbial biosynthesis or direct biochemical conversion. Apparent compositional changes may instead reflect altered extractability and enhanced analytical accessibility rather than formation of novel metabolites [21,70]. Increased detectability may additionally result from liberation from insoluble fractions or shifts in compound partitioning during fermentation [57,87].

Interpretation of matrix-bound metabolite release therefore requires particular methodological caution because fermentation simultaneously modifies substrate structure, microbial activity, extraction efficiency, and analytical detectability. In complex food matrices, assigning compositional changes to a single biological mechanism frequently remains difficult without targeted metabolomic or mechanistic validation [24,68]. Emerging systems-level approaches increasingly emphasize the importance of resolving strain-specific metabolic contributions within broader matrix-dependent interactions [94].

Current evidence nevertheless suggests that fermentation-associated restructuring of food matrices may represent an additional dimension of yeast functionality extending beyond conventional fermentation outcomes. Importantly, these effects appear to emerge through interactions among strain-specific metabolism, enzymatic activity, and matrix composition rather than through universally predictable species-level behavior [2,3].

Taken together, the emerging evidence discussed above suggests that yeast functionality in food fermentations may extend beyond conventional technological outputs and involve broader compositional restructuring processes. However, because these effects remain highly context-dependent and frequently mechanistically unresolved, interpretation requires careful distinction between direct microbial transformation and fermentation-associated matrix effects [1,3]. An overview of the proposed mechanisms and their associated interpretation limitations is presented in Table 3.

**Table 3:** Proposed mechanisms underlying yeast-mediated transformation of food-associated bioactive compounds during fermentation. The overview summarizes representative mechanisms through which non-*Saccharomyces* yeasts may influence compositional restructuring of food-associated metabolites, together with their potential technological relevance and key interpretational limitations affecting mechanistic attribution.

Proposed Mechanism	Target Compound(s)	Proposed Microbial Action	Potential Technological Relevance	Major Interpretational Limitation	Key Validation Requirement	Representative References
Glycoside cleavage (β-glucosidase activity)	Glycosides, terpene precursors, aroma-associated conjugates	Hydrolysis of glycosidic bonds releasing free volatile or bioactive compounds	Aroma enhancement, liberation of bound metabolites, improved sensory complexity	Strongly strain-dependent; enzyme expression affected by glucose repression, pH, and ethanol	Confirmation of enzymatic activity under fermentation-relevant conditions	[21,31]
Phenolic transformation	Phenolic acids, flavonoids, conjugated phenolics	Hydrolysis, oxidation–reduction reactions, esterase activity, decarboxylation	Modification of phenolic profile and potential bioactive accessibility	Difficult to distinguish true biotransformation from matrix release or analytical effects	Targeted metabolomic or mechanistic validation	[18,70]
Terpene liberation	Glycosidically bound terpenes, aroma precursors	β-glucosidase- and esterase-mediated release of volatile compounds	Increased floral, citrus, and fruity aroma characteristics	Strong precursor dependency and matrix specificity	Validation of precursor availability and volatile release	[29,30]
Adsorption to yeast biomass	Phenolics, volatile compounds, matrix-associated metabolites	Binding to mannoproteins and cell wall components	Altered sensory or compositional profile	Apparent concentration changes may not reflect true metabolism	Differentiation between adsorption and metabolic conversion	[46,70]
Matrix-associated metabolite release	Matrix-bound phenolics, glycosides, peptides, terpenes	Fermentation-associated restructuring of food matrix enhancing extractability	Increased accessibility of bioactive or aroma-associated compounds	Difficult to separate microbial effects from physicochemical release	Extraction/accessibility controls during fermentation	[18,59]
Microbial redox-mediated modification	Phenolics, aroma compounds, redox-sensitive metabolites	Oxidation–reduction reactions during fermentation	Potential compositional restructuring and sensory modulation	Mechanisms remain poorly resolved and strain-specific	Strain-level mechanistic validation	[24,68]

## 7. Toward a Matrix–Trait–Function Framework for Predictive Yeast Selection

### 7.1. Why Current Selection Strategies Remain Limited

Despite the growing diversity of non-*Saccharomyces* applications in food fermentation, yeast selection strategies frequently remain guided by broad taxonomic assumptions or isolated technological objectives rather than by integrated functional reasoning. In practice, microbial selection is often simplified through descriptors such as “aroma-enhancing yeast”, “acidifying yeast”, “low-alcohol yeast”, or “bioprotective strain”, implicitly assuming a level of functional consistency that may not adequately reflect biological and technological complexity [3]. Increasing evidence suggests that fermentation-relevant performance frequently varies at the strain level and emerges through interactions among microbial metabolism, matrix composition, and process conditions rather than through species identity alone [2,24]. Consequently, reliance on taxonomy-centered or single-trait descriptions may limit predictive applicability across food systems and fermentation contexts [6].

Importantly, similar technological outcomes may be achieved through distinct metabolic strategies, whereas the same microbial trait may generate contrasting consequences depending on matrix composition and product objectives [25,50]. For example, restricted maltose utilization may represent a technological limitation in conventional brewing while becoming advantageous in low- and non-alcoholic systems where ethanol reduction is desirable [5,82]. Similarly, strong ester production may enhance sensory complexity in wine or fruit fermentations yet prove undesirable in products requiring restrained flavor expression or compositional neutrality [30,41]. Enzymatic hydrolysis of glycosidically bound compounds may further exhibit substantial technological relevance in terpene-rich plant matrices while contributing little in substrates lacking suitable precursor compounds [18,31]. These examples illustrate that fermentation functionality acquires technological significance only within specific matrix contexts [51].

Microbial performance additionally reflects ecological interactions extending beyond individual strain characteristics. Nutrient competition, oxygen availability, microbial succession, co-fermentation strategies, and interactions with indigenous microbiota may substantially reshape fermentation trajectories and final product outcomes [77,93]. Fermentation functionality should therefore be interpreted as an emergent property arising through matrix–microbe–process interactions rather than as a fixed microbial characteristic predictable solely from taxonomic identity [24,97].

Collectively, these limitations highlight the need for a more predictive and application-oriented framework capable of integrating microbial functionality with food matrix characteristics and technological objectives. Rather than asking “Which species should be used?”, a functionality-oriented perspective instead asks “Which metabolic traits are required under specific matrix constraints to achieve the desired technological outcome?” [2]. This shift from taxonomy-centered toward functionality-driven reasoning may provide a more informative basis for targeted yeast selection and more reproducible fermentation design in modern food systems [1,3].

### 7.2. Proposed Matrix–Trait–Function Framework

Based on the evidence synthesized throughout this review, a functionality-oriented interpretation of yeast diversity may benefit from a conceptual framework integrating food matrix characteristics, microbial metabolic traits, and desired technological outcomes. Rather than representing a universal predictive model, the proposed framework is intended as a decision-support perspective for organizing current knowledge regarding context-dependent yeast functionality in food fermentation systems [3]. In contrast to conventional taxonomy-centered selection strategies, which frequently prioritize species designation or isolated technological traits, the matrix–trait–function framework emphasizes how fermentation outcomes emerge through interactions among microbial metabolism, substrate composition, and process objectives [2,24].

Within this framework, yeast selection begins not with species identity but with definition of the desired product objective together with major matrix-specific constraints. Product objectives may include reduced ethanol formation, aroma enhancement, biological acidification, microbial stabilization, texture modulation, or targeted metabolite transformation [6,30]. Simultaneously, food matrices impose distinct physicochemical limitations, including carbohydrate composition, nitrogen availability, pH, oxygen transfer, phenolic content, osmotic pressure, microbial ecology, and precursor abundance [25,97]. These factors collectively define the selective environment in which microbial traits are expressed and determine whether specific metabolic capabilities become technologically relevant [77,93].

Subsequently, microbial selection may be guided through identification of functional traits required to achieve the targeted fermentation outcome. Depending on the application, relevant traits may include restricted maltose utilization, respiratory metabolism, ester biosynthesis, acidification capacity, enzymatic hydrolysis of conjugated metabolites, microbial competitiveness, or stress tolerance [5,31]. Importantly, similar technological outcomes may emerge through distinct metabolic strategies, whereas the same microbial trait may generate contrasting consequences depending on matrix composition and fermentation objectives [1,2]. For example, limited fermentative performance may represent either a technological constraint or an advantage depending on whether the objective is complete sugar conversion or controlled ethanol restriction [5,82]. Likewise, enzymatic activities associated with glycoside cleavage may exhibit substantial technological relevance in terpene-rich botanical matrices while contributing minimally in substrates lacking suitable precursor compounds [18,31].

Importantly, the proposed framework further recognizes that fermentation outcomes frequently involve trade-offs rather than isolated technological benefits. Enhanced aroma complexity may coincide with excessive higher alcohol formation or sensory imbalance [30,69]. Biological acidification may improve microbial stability while simultaneously reshaping flavor perception and product acceptability [50]. Likewise, microbial competitiveness may influence co-fermentation dynamics, whereas metabolite transformation frequently remains difficult to predict under industrial conditions because of strain-level and process-related variability [24,77]. Functionality-oriented yeast selection should therefore be interpreted as a context-dependent optimization process balancing desired technological performance with matrix-specific limitations and process feasibility [2].

Although predictive applicability remains constrained by pronounced strain-level variability and incomplete mechanistic understanding, integrating microbial functionality with matrix composition may provide a more informative basis for guiding research and industrial implementation than species designation alone [1,3]. Consequently, the matrix–trait–function framework proposed here may support more targeted, reproducible, and application-oriented exploitation of yeast diversity in modern food fermentation systems [2].

## 8. Limitations, Challenges, and Future Perspectives

### 8.1. Strain Variability and Reproducibility Challenges

Despite rapidly expanding interest in *non-Saccharomyces* yeasts, one of the principal limitations constraining predictive implementation in food fermentation systems remains pronounced strain-level variability within individual species. Fermentation-relevant characteristics, including sugar utilization, aroma formation, acidification capacity, enzymatic activity, stress tolerance, microbial competitiveness, and metabolite transformation, frequently differ substantially among strains belonging to the same taxonomic group [3,23].

As a result, fermentation performance often reflects strain-specific metabolic behavior rather than species designation alone, limiting the predictive applicability of broad taxonomic classifications [2,24]. Species-level descriptions may therefore provide only limited guidance regarding actual fermentation performance under industrially relevant conditions [6].

Reproducibility is further complicated by strong environmental sensitivity of microbial metabolism and the context-dependent nature of functional trait expression. Variations in sugar composition, nitrogen availability, oxygen transfer, pH, precursor abundance, microbial ecology, and processing conditions may substantially reshape fermentation trajectories even when the same strain is applied [25,97]. Environmental factors have repeatedly been shown to influence microbial metabolism, stress responses, and expression of functional traits, thereby affecting both technological performance and reproducibility [77,93]. Such context dependence complicates extrapolation of laboratory-scale findings toward industrial implementation and may partly explain inconsistencies frequently reported across studies investigating similar microbial systems [41,50].

Another important challenge concerns methodological heterogeneity in characterization of microbial functionality. Differences in inoculation strategy, fermentation scale, substrate composition, analytical methodology, process control, co-culture design, and reporting practices frequently complicate direct cross-study comparability [2,30]. These limitations become particularly relevant when evaluating aroma formation, microbial interactions, or fermentation-associated metabolite transformation, where methodological variability may substantially influence reported outcomes and mechanistic interpretation [18,21]. Consequently, improving reproducibility may require stronger methodological harmonization, strain-level characterization, and matrix-specific validation to enhance translational relevance of future research [1,84].

### 8.2. Regulatory, Safety, and Industrial Translation Challenges

Although many non-*Saccharomyces* yeasts demonstrate promising technological potential, broader industrial adoption remains influenced by safety assessment, regulatory acceptance, process robustness, and manufacturing feasibility. Species-specific safety status, including GRAS (Generally Recognized as Safe) and QPS (Qualified Presumption of Safety) designation, may substantially influence practical implementation across food sectors and determine whether particular strains can be integrated into commercial fermentation systems [13,14]. Regulatory acceptance may additionally differ according to food category, intended application, and regional legislative frameworks, thereby influencing translational feasibility beyond technological performance alone [15].

Importantly, microbial performance demonstrated under laboratory conditions may not consistently translate to industrial fermentation environments characterized by larger-scale variability, altered oxygen transfer, microbial competition, fluctuating nutrient availability, and increased process complexity [2,3]. Scale-up frequently introduces additional sources of variability capable of reshaping microbial behavior and fermentation outcomes, particularly in mixed-culture systems or matrices containing complex indigenous microbiota [77,93]. Consequently, fermentation performance observed under controlled experimental conditions should be interpreted cautiously when predicting industrial robustness and reproducibility [24].

Moreover, microbial functionality frequently involves trade-offs requiring careful technological optimization. Enhanced aroma formation may coincide with undesirable sensory deviations, excessive volatile production, or flavor imbalance depending on matrix composition and process objectives [30,69]. Biological acidification may improve microbial stability while simultaneously altering sensory perception and product acceptability [50]. Likewise, microbial competitiveness may reshape fermentation trajectories in unintended ways, particularly during co-fermentation or spontaneous fermentation systems [77].

Similarly, enzymatic transformation of food-associated metabolites may remain difficult to predict across different matrices due to pronounced strain variability, precursor availability, and incomplete mechanistic understanding [18,31]. Because microbial functionality frequently emerges through interactions among matrix composition, metabolic traits, and process conditions, future industrial implementation will likely require more systematic evaluation of matrix–trait compatibility rather than reliance on broad species-level assumptions [2,25].

### 8.3. Future Perspectives: Toward Predictive and Precision-Oriented Fermentation

Despite the rapidly growing diversity of reported food-associated non-*Saccharomyces* yeasts, current approaches to strain selection frequently remain constrained by taxonomy-centered assumptions and empirical screening strategies. In many fermentation systems, microbial selection continues to rely primarily on species identity or isolated technological traits, thereby limiting reproducibility and reducing transferability of observed functionality across different food matrices and process conditions [3,6]. Increasing evidence synthesized throughout this review suggests that future progress in food fermentation may require moving beyond descriptive species-based classification toward a more predictive understanding of strain-specific functionality under defined matrix constraints [2,24].

Rather than asking which yeast species are generally associated with desirable technological outcomes, future approaches may increasingly focus on identifying which strain-specific metabolic traits are most likely to generate targeted functionality under specific physicochemical and ecological conditions. Such a shift may support transition toward precision-oriented fermentation design, in which microbial selection is guided by compatibility among desired product objectives, food matrix composition, and microbial metabolic potential rather than taxonomy alone [24]. Emerging industrial perspectives increasingly emphasize predictive and systems-based approaches for improving fermentation reproducibility and technological control across diverse food matrices [16].

This perspective further supports development of predictive fermentation ecology, where fermentation outcomes are interpreted as emergent properties resulting from interactions among microbial physiology, substrate composition, oxygen transfer, nutrient availability, microbial competition, and process conditions [14]. Importantly, similar technological outcomes may emerge through distinct metabolic routes, while identical microbial traits may generate divergent consequences depending on matrix characteristics and processing objectives [2,17]. Consequently, future research may increasingly benefit from experimental designs integrating strain-level validation across multiple food matrices rather than relying on isolated laboratory observations or broad species-level generalizations [23].

Emerging analytical tools, including genomics, metabolomics, transcriptomics, systems biology, and computational modeling, may further improve mechanistic understanding of yeast functionality [68,98]. However, their value will likely depend less on generating increasingly descriptive datasets and more on improving interpretation of how microbial metabolic traits translate into reproducible technological outcomes under realistic fermentation conditions [67]. Future progress may therefore require stronger integration between molecular-scale observations and fermentation-relevant performance metrics, particularly regarding aroma biogenesis, metabolite transformation, microbial interactions, and matrix-dependent metabolic regulation [68].

A major challenge for future industrial implementation remains the persistent gap between laboratory-scale observations and predictable performance in commercial fermentation environments. Although many strains demonstrate promising functionality under controlled experimental conditions, reproducibility frequently declines under industrially relevant conditions because of matrix complexity, microbial competition, process variability, and environmental heterogeneity [2,10]. Bridging this translational gap will likely require more systematic validation of matrix–trait compatibility together with improved methodological harmonization and standardized reporting of fermentation conditions and strain performance [30].

Ultimately, the future of functional yeast diversity in food fermentations may depend less on expanding taxonomic catalogues and more on developing a predictive understanding of how strain-specific metabolic traits interact with food matrices to generate reproducible and technologically meaningful outcomes. In this context, the matrix–trait–function framework proposed in the present review may provide a conceptual basis for transitioning from empirical yeast screening toward more mechanistically informed, reproducible, and application-oriented strategies for fermentation design [1,3].

## 9. Conclusions

The growing diversity of food fermentation systems increasingly challenges taxonomy-centered approaches to understanding and exploiting non-*Saccharomyces* yeasts. As highlighted throughout this review, fermentation functionality rarely emerges from species identity alone but instead reflects interactions among strain-specific metabolic traits, food matrix composition, environmental conditions, and technological objectives. Consequently, broad species-level descriptors frequently provide limited predictive value across different food systems and processing environments. By moving beyond predominantly taxonomy-centered interpretation, this review examined non-*Saccharomyces* yeasts through a matrix–trait–function perspective, emphasizing microbial functionality as a context-dependent and strain-specific property rather than a fixed species-associated characteristic. Evidence synthesized across diverse fermentation systems further demonstrates that food matrix composition fundamentally shapes microbial performance, technological outcomes, and fermentation-associated metabolite transformation. The proposed matrix–trait–function framework provides a more predictive and application-oriented perspective for targeted yeast selection by integrating microbial metabolic capabilities with matrix-specific constraints and technological objectives. Future progress in functional yeast applications will likely depend less on expanding taxonomic catalogues and more on improving predictive understanding of strain-specific functionality to support more reproducible and precision-oriented fermentation design.

## Figures and Tables

**Table 1:** Representative example of strain-associated functional traits of selected food-associated non-*Saccharomyces* yeasts across food fermentation systems. The overview summarizes commonly reported technological tendencies rather than universally transferable species-level characteristics, emphasizing the context-dependent nature of yeast functionality.

Yeast Species	Representative Strain-Associated Functional Trait(s)	Relevant Food Matrix	Typical Technological Objective	Major Matrix- or Strain-Dependent Limitation	Representative References
*Torulaspora* *delbrueckii*	Ester biosynthesis, aroma modulation, moderate ethanol reduction	Beer, wine, fruit fermentations	Enhanced fruity/floral aroma, increased sensory complexity	Strong strain-level variability; outcomes influenced by nitrogen availability, temperature, and inoculation strategy	[5,26]
*Lachancea* *thermotolerans*	Biological acidification via lactic acid production, aroma modulation	Wine, brewing systems	Increased acidity, improved freshness, microbial stabilization	Acidification strongly strain- and matrix-dependent; may alter sensory balance	[23,28]
*Metschnikowia pulcherrima*	Restricted sugar utilization, microbial antagonism, aroma modulation	Brewing, wine, fruit beverages	Controlled ethanol reduction, aroma diversification, bioprotection	Limited maltose utilization may impair attenuation; highly strain-dependent fermentation performance	[22,29]
*Pichia kluyveri*	Thiol and terpene release, ester formation	Wine, hop-containing fermentations	Enhanced tropical and fruity aroma expression	Strong precursor dependency; enzymatic activity varies substantially across strains and matrices	[30,31]
*Hanseniaspora uvarum*	Aroma diversification, ester production	Wine, fruit fermentations	Increased aroma complexity	Limited fermentation robustness; volatile production strongly condition-dependent	[23,32]
*Cyberlindnera saturnus*	Restricted fermentative capacity	Brewing, low-alcohol beverages	Controlled ethanol reduction	Strain-dependent sugar utilization and incomplete attenuation risk	[8,33]
*Wickerhamomyces* *anomalus*	Bioprotection, microbial antagonism	Cereal, bakery, beverages	Suppression of spoilage organisms, microbial stabilization	Excessive antagonism may interfere with desirable starter cultures	[34,35]
*Pichia* spp.	Killer toxin production, antagonistic activity	Fermented foods, beverages	Microbial stabilization	Antimicrobial effects remain strongly strain- and environment-dependent	[7,36]

**Table 2:** Representative matrix–trait–function relationships governing non-*Saccharomyces* yeast performance across major food fermentation systems. The overview summarizes how food matrix composition interacts with strain-associated microbial traits to shape technological functionality, highlighting the context-dependent and application-oriented nature of yeast performance.

Food Matrix	Major Matrix Characteristics/Constraints	Key Relevant Microbial Traits	Targeted Technological Outcomes	Major Context-Dependent Limitation	Representative References
Brewing systems	Maltose- and maltotriose-rich substrate, hop-derived antimicrobial compounds, low oxygen availability, defined FAN composition	Restricted sugar utilization, ester biosynthesis, glycerol production, β-glucosidase activity, stress tolerance	Low-/non-alcoholic beer production, aroma enhancement, ethanol modulation, sensory diversification	Limited attenuation, strong strain dependence, precursor availability, co-fermentation effects	[8,22,23]
Wine and fruit fermentations	Glucose- and fructose-rich substrate, acidic pH, high osmotic pressure, terpene/glycoside-rich matrices	Ester biosynthesis, β-glucosidase activity, thiol release, acidification, stress adaptation	Aroma enhancement, acidity modulation, increased sensory complexity	Strong influence of grape/fruit variety, nutrient composition, fermentation temperature, precursor abundance	[5,26]
Cereal and bakery fermentations	Starch-derived substrates, limited fermentable sugars, LAB co-occurrence, variable nutrient accessibility	Enzymatic hydrolysis, microbial interactions, stress tolerance, aroma generation	Texture development, aroma diversification, dough functionality	High ecological complexity; outcomes strongly depend on microbial consortia	[10,59]
Plant-based fermented foods	High phenolic content, glycosides, structurally complex plant matrices, variable nutrient availability	β-glucosidase activity, esterase activity, metabolite transformation, acidification	Functional enhancement, aroma release, compositional restructuring	Low reproducibility and incomplete mechanistic understanding	[18,31]
Functional beverages/low-alcohol systems	Reduced sugar content, altered nutrient composition, sensory instability risk	Restricted carbon metabolism, glycerol production, aroma modulation, microbial stabilization	Ethanol reduction, mouthfeel enhancement, sensory balancing	Trade-offs between aroma, sweetness, body, and microbial robustness	[22,33]

## Data Availability

No new data were generated or analyzed in this study. Data sharing is not applicable to this article.

## Abbreviations

The following abbreviations are used in this manuscript:

FAN	Free Amino Nitrogen
GRAS	Generally Recognized as Safe
LAB	Lactic Acid Bacteria
QPS	Qualified Presumption of Safety
